# Duties of the Reserve Physician in Case of Mobilization

**Published:** 1914-09

**Authors:** 


### ABSTRACTS

---

**Duties of the Reserve Physician in Case of Mobilization,** Le Progres-Med., Aug. 1, 1914. Every physician has an order of mobilization indicating his assignment. He must inform himself of the departure of trains, personally supervise the loading of his baggage and equipment, secure passage in the compartment to which his rank entitles him, or, if such compartments are full, take any other place available. According to rank, from three trunks down to half of a trunk are allowed. These are supplied by the government and measure

65x22x38 c. m. and weigh, empty, 7.6 to 7.8 kilos. When packed, they must not exceed 17 kilos except that a slight excess is allowed for majors of the second class entitled to two trunks. This corresponds to the following outfit for a trunk: 1 pair of trousers, 1 tunic, 1 pair of shoes, 4 pairs of socks, 3 sets of underwear and shirts, I police helmet, 6 handkerchiefs, 6 collars, 3 towels, 2 wash cloths, 1 cup, 1 tooth brush, clothes brush, etc. Table utensils are supplied in cases for parties of six. The uniform, etc., are prescribed and officers of sufficient rank are furnished with a horse and equipment, the last not to exceed 150 francs. Among the requirements are an identification card on a cord. Physicians report first to the commandant of the place indicated, than to the chief medical officer of their assignment. At the place of mobilization, the physician draws his indemnity of enlistment amounting to 700 to 1,500 francs according to rank. The pay for an aide-major of the second class (corresponding somewhat to our rank of captain on the medical staff) is 6.70 francs a day. Half of this can be assigned to a wife, parent or child, a quarter to another person. During the period of concentration, the physician learns the duties of his office, familiarizes himself with the assistants, etc.

